# Two-dimensional dysprosium(III) triiodate(V) dihydrate, Dy(IO_3_)_3_(H_2_O)·H_2_O

**DOI:** 10.1107/S1600536809027068

**Published:** 2009-07-15

**Authors:** Wenxiang Chai, Li Song, Hongsheng Shi, Laishun Qin, Kangying Shu

**Affiliations:** a College of Materials Science and Engineering, China Jiliang University, Hangzhou 310018, People’s Republic of China; b Department of Chemistry, Key Laboratory of Advanced Textile Materials and Manufacturing Technology of the Education Ministry, Zhejiang Sci-Tech University, Hangzhou 310018, People’s Republic of China

## Abstract

During our research into novel nonlinear optical materials using 1,10-phenanthroline as an appending ligand on lanthanide iodates, crystals of an infinite layered Dy^III^ iodate compound, Dy(IO_3_)_3_(H_2_O)·H_2_O, were obtained under hydro­thermal conditions. The Dy^III^ cation has a dicapped trigonal prismatic coordination environment consisting of one water O atom and seven other O atoms from seven iodate anions. These iodate anions bridge the Dy^III^ cations into a two-dimensional structure. Through O—H⋯O hydrogen bonds, all of these layers stack along [111], giving a supra­molecular channel, with the solvent water mol­ecules filling the voids.

## Related literature

For related materials with non-linear optical propertie, see: Rosenzweig & Morosin (1966 ▶); Liminga *et al.* (1977 ▶); Ok & Halasyamani (2005 ▶). The method of preparation was based on HIO_3_, which is different to the previous method of obtaining periodates (Douglas *et al.*, 2004 ▶; Assefa *et al.*, 2006 ▶). For noncentrosymmetric inorganic–organic framework structures synthesized from organic ligands, see: Sun *et al.* (2009 ▶). For related structrues, see: Sun *et al.* (2009 ▶); Assefa *et al.* (2006 ▶); Douglas *et al.* (2004 ▶); Ok & Halasyamani (2005 ▶); Chen *et al.* (2005 ▶).

## Experimental

### 

#### Crystal data

Dy(IO_3_)_3_H_2_O·H_2_O
*M* *_r_* = 723.23
Triclinic, 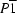

*a* = 7.15990 (10) Å
*b* = 7.4292 (1) Å
*c* = 10.64430 (10) Å
α = 95.161 (12)°
β = 104.858 (7)°
γ = 110.081 (8)°
*V* = 504.00 (5) Å^3^
*Z* = 2
Mo *K*α radiation
μ = 16.65 mm^−1^
*T* = 293 K
0.16 × 0.12 × 0.06 mm

#### Data collection

Rigaku R-AXIS RAPID diffractometer
Absorption correction: multi-scan (*ABSCOR*; Higashi, 1995 ▶) *T*_min_ = 0.136, *T*_max_ = 0.435 (expected range = 0.115–0.368)
3819 measured reflections
2260 independent reflections
2067 reflections with *I* > 2σ(*I*)
*R*_int_ = 0.027

#### Refinement

*R*[*F* ^2^ > 2σ(*F* ^2^)] = 0.038
*wR*(*F* ^2^) = 0.109
*S* = 1.06
2260 reflections
141 parameters
2 restraints
H atoms treated by a mixture of independent and constrained refinement
Δρ_max_ = 2.79 e Å^−3^
Δρ_min_ = −3.20 e Å^−3^

### 

Data collection: *PROCESS-AUTO* (Rigaku, 1998 ▶); cell refinement: *PROCESS-AUTO*; data reduction: *CrystalStructure* (Rigaku/MSC, 2004 ▶); program(s) used to solve structure: *SHELXS97* (Sheldrick, 2008 ▶); program(s) used to refine structure: *SHELXL97* (Sheldrick, 2008 ▶) and *PLATON* (Spek, 2009 ▶; van der Sluis & Spek, 1990 ▶); molecular graphics: *SHELXTL* (Sheldrick, 2008 ▶); software used to prepare material for publication: *SHELXTL*.

## Supplementary Material

Crystal structure: contains datablocks I, global. DOI: 10.1107/S1600536809027068/br2111sup1.cif

Structure factors: contains datablocks I. DOI: 10.1107/S1600536809027068/br2111Isup2.hkl

Additional supplementary materials:  crystallographic information; 3D view; checkCIF report

## Figures and Tables

**Table 1:** Hydrogen-bond geometry (Å, °)

*D*—H⋯*A*	*D*—H	H⋯*A*	*D*⋯*A*	*D*—H⋯*A*
O10—H10*A*⋯O3^i^	0.80	2.29	2.873 (10)	131
O10—H10*B*⋯O9^i^	0.80	2.33	2.753 (11)	114
O11—H11*A*⋯O8^ii^	0.80	2.22	2.954 (11)	153
O11—H11*B*⋯O7^iii^	0.80	2.26	2.946 (11)	145

Symmetry codes: (i) 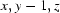; (ii) 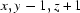; (iii) 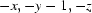.

**Fig. 1.**
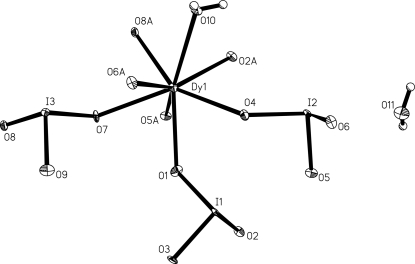
Structure and labeling of the title compound, with displacement ellipsoids drawn at the 30% probability level and H atoms shown as small spheres of arbitrary radii.

**Fig. 2.**
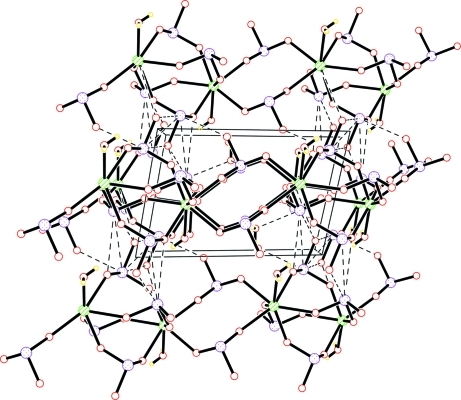
The packing diagram viewed along the a-direction, Dy: green diagonal; I: purple inner dot; O: red; and H: small blue circles. And hydrogen bonds are denoted as dash lines.

## supplementary crystallographic information

### Comment

In the 1970s, metal iodates have been extensively studied by Bell Laboratories not only for their nonlinear optical (NLO) properties but also for ferroelectric, piezoelectric and pyroelectric properties (Rosenzweig & Morosin, 1966; Liminga *et al.*, 1977). In attempts to prepare noncentrosymmetric structures of lanthanide iodates, about six anhydrous structure types have been reported, in addition to numerous hydrated structures ranging from hemihydrates to pentahydrates. (Assefa *et al.*, 2006). After comparing these structure types, herein, we find that the hydrated structures favor of adopting centrosymmetric structures. Then organic ligands are come into our view because they could form noncentrosymmetric inorganic–organic framework structures with metal ion. (Sun *et al.*, 2009). Here, we firstly report a infinite layered Dy^III^ iodate dihydrate synthesized from the hydrothermal reaction of Dy_2_O_3_, HIO_3_ and 1,10-phenanthroline.

In the title compound, the Dy^III^ cation has dicapped trigonal prismatic coordination sphere. The coordination enciroments of the rare earth Dy^III^ cation consist of eight O atoms derived from seven iodate anions and one water molecule (see Fig. 1). And these seven iodates are classed two types, one is three 3-connected iodates (of I2) through three O atoms, and the other is four iodates 2-connected (of I1 or I3) through two O atoms. Then these iodate anions bridge Dy atoms into two dimensional structure. And between the adjacent layers, there are two types of hydrogen bonds, one is O10—H10A···O3 bond, the other is O10—H10B···O9 bond. Then through these hydrogen bonds, all of these layers stacking along [111] axis to give out of a supramolecular channel. And the solvent water molecules fill in the channels, and stick on the channel with two hydrogen bonds of O11—H11A···O8 and O11—H11B···O7. (see Fig. 2) The hydrogen bonding data of lengths and angles are in the range of ordinary examples and have been examined by the *PLATON* program (Spek, 2009; van der Sluis & Spek, 1990).

### Experimental

All chemicals were obtained from commercial sources and were used as received. The title compound was handily synthesized by a hydrothermal reaction from iodic acid. To a 25 ml stainless steal Teflon-lined reaction vessel, Dy_2_O_3_ (0.2 mmol, 75 mg), HIO_3_ (0.8 mmol, 141 mg), 1,10-phenanthroline (0.4 mmol, 80 mg) and 13 ml H_2_O were added and stirred thoroughly for 1 h, then heated at 393 K for 2 d. After cooling down to room temperature, some colorless crystalline product (I) was obtained.

### Refinement

The structure was solved using direct methods and refined by full-matrix least-squares techniques. All non-hydrogen atoms were assigned anisotropic displacement parameters in the refinement. All H atoms were added at calculated positions and refined using a riding model.(Sheldrick, 2008). The maximum (2.79) and minumum (-3.20) in the difference electron density were found at 0.0198 0.3244 0.7024 [1.01 Å from DY1] and 0.2071 0.4512 0.7963 [0.60 Å from DY1], respectively.

The O6 has ADP max/min ratio 6.70. This result may be due to the packing of supramolecule.

### Crystal data

**Table:**

Dy(IO_3_)_3_H_2_O·H_2_O	*Z* = 2
*M**_r_* = 723.23	*F*(000) = 634
Triclinic, *P*1	*D*_x_ = 4.766 Mg m^−^^3^
Hall symbol: -P 1	Mo *K*α radiation, λ = 0.71075 Å
*a* = 7.1599 (1) Å	Cell parameters from 1561 reflections
*b* = 7.4292 (1) Å	θ = 2.0–27.5°
*c* = 10.6443 (1) Å	µ = 16.65 mm^−^^1^
α = 95.161 (12)°	*T* = 293 K
β = 104.858 (7)°	Block, colourless
γ = 110.081 (8)°	0.16 × 0.12 × 0.06 mm
*V* = 504.00 (5) Å^3^	

### Data collection

**Table:**

Rigaku R-AXIS RAPID diffractometer	2260 independent reflections
Radiation source: fine-focus sealed tube	2067 reflections with *I* > 2σ(*I*)
graphite	*R*_int_ = 0.027
Detector resolution: 14.6306 pixels mm^-1^	θ_max_ = 27.5°, θ_min_ = 3.2°
CCD profile fitting scans	*h* = −9→9
Absorption correction: multi-scan (*ABSCOR*; Higashi, 1995)	*k* = −7→9
*T*_min_ = 0.136, *T*_max_ = 0.435	*l* = −13→13
3819 measured reflections	

### Refinement

**Table:**

Refinement on *F*^2^	Secondary atom site location: difference Fourier map
Least-squares matrix: full	Hydrogen site location: inferred from neighbouring sites
*R*[*F*^2^ > 2σ(*F*^2^)] = 0.038	H atoms treated by a mixture of independent and constrained refinement
*wR*(*F*^2^) = 0.109	*w* = 1/[σ^2^(*F*_o_^2^) + (0.0647*P*)^2^ + 5.3292*P*] where *P* = (*F*_o_^2^ + 2*F*_c_^2^)/3
*S* = 1.06	(Δ/σ)_max_ < 0.001
2260 reflections	Δρ_max_ = 2.79 e Å^−^^3^
141 parameters	Δρ_min_ = −3.20 e Å^−^^3^
2 restraints	Extinction correction: *SHELXL97* (Sheldrick, 2008), Fc^*^=kFc[1+0.001xFc^2^λ^3^/sin(2θ)]^-1/4^
Primary atom site location: structure-invariant direct methods	Extinction coefficient: 0.0126 (8)

### Special details

**Table:**

Geometry. All e.s.d.'s (except the e.s.d. in the dihedral angle between two l.s. planes) are estimated using the full covariance matrix. The cell e.s.d.'s are taken into account individually in the estimation of e.s.d.'s in distances, angles and torsion angles; correlations between e.s.d.'s in cell parameters are only used when they are defined by crystal symmetry. An approximate (isotropic) treatment of cell e.s.d.'s is used for estimating e.s.d.'s involving l.s. planes.
Refinement. Refinement of *F*^2^ against ALL reflections. The weighted *R*-factor *wR* and goodness of fit *S* are based on *F*^2^, conventional *R*-factors *R* are based on *F*, with *F* set to zero for negative *F*^2^. The threshold expression of *F*^2^ > σ(*F*^2^) is used only for calculating *R*-factors(gt) *etc*. and is not relevant to the choice of reflections for refinement. *R*-factors based on *F*^2^ are statistically about twice as large as those based on *F*, and *R*- factors based on ALL data will be even larger.

### Fractional atomic coordinates and isotropic or equivalent isotropic displacement parameters (Å^2^)

**Table:**

	*x*	*y*	*z*	*U*_iso_*/*U*_eq_	
Dy1	0.11491 (7)	−0.58338 (7)	−0.21096 (4)	0.01105 (18)	
I1	0.30890 (8)	−0.14149 (8)	0.07303 (5)	0.00659 (18)	
I2	0.28110 (8)	−0.63445 (8)	0.16834 (5)	0.00652 (18)	
I3	0.27848 (9)	−0.26870 (9)	−0.45631 (6)	0.00862 (19)	
O1	0.1496 (11)	−0.2711 (12)	−0.0929 (7)	0.0178 (15)	
O2	0.0966 (11)	−0.1450 (11)	0.1365 (7)	0.0144 (14)	
O3	0.3778 (11)	0.1033 (10)	0.0380 (7)	0.0134 (14)	
O4	0.2940 (11)	−0.5350 (11)	0.0209 (7)	0.0155 (15)	
O5	0.2291 (10)	−0.4405 (11)	0.2502 (7)	0.0131 (14)	
O6	0.5556 (10)	−0.5510 (11)	0.2555 (7)	0.0121 (14)	
O7	0.0908 (11)	−0.3710 (11)	−0.3699 (7)	0.0130 (14)	
O8	0.1065 (11)	−0.1986 (11)	−0.5813 (7)	0.0125 (14)	
O9	0.4335 (11)	−0.0352 (12)	−0.3515 (8)	0.0187 (16)	
O10	0.2235 (12)	−0.8611 (12)	−0.2319 (8)	0.0185 (16)	
H10A	0.198 (15)	−0.928 (9)	−0.179 (7)	0.028*	
H10B	0.346 (4)	−0.8277 (13)	−0.221 (10)	0.028*	
O11	0.2419 (13)	−0.7837 (13)	0.3943 (9)	0.0257 (18)	
H11A	0.225 (3)	−0.897 (15)	0.3811 (19)	0.039*	
H11B	0.144 (13)	−0.7725 (18)	0.412 (2)	0.039*	

### Atomic displacement parameters (Å^2^)

**Table:**

	*U*^11^	*U*^22^	*U*^33^	*U*^12^	*U*^13^	*U*^23^
Dy1	0.0094 (3)	0.0136 (3)	0.0131 (3)	0.00658 (19)	0.00455 (18)	0.00444 (19)
I1	0.0048 (3)	0.0061 (3)	0.0105 (3)	0.0031 (2)	0.0028 (2)	0.0041 (2)
I2	0.0040 (3)	0.0067 (3)	0.0111 (3)	0.0040 (2)	0.0027 (2)	0.0039 (2)
I3	0.0076 (3)	0.0109 (3)	0.0101 (3)	0.0063 (2)	0.0032 (2)	0.0026 (2)
O1	0.014 (3)	0.023 (4)	0.012 (3)	0.010 (3)	−0.004 (3)	−0.002 (3)
O2	0.011 (3)	0.014 (4)	0.022 (4)	0.006 (3)	0.009 (3)	0.007 (3)
O3	0.020 (3)	0.006 (3)	0.020 (4)	0.007 (3)	0.011 (3)	0.006 (3)
O4	0.019 (4)	0.013 (4)	0.014 (3)	0.006 (3)	0.005 (3)	0.006 (3)
O5	0.006 (3)	0.014 (4)	0.021 (4)	0.005 (3)	0.007 (3)	0.002 (3)
O6	0.001 (3)	0.016 (4)	0.018 (3)	0.002 (3)	0.003 (3)	0.007 (3)
O7	0.014 (3)	0.020 (4)	0.013 (3)	0.012 (3)	0.007 (3)	0.013 (3)
O8	0.014 (3)	0.013 (4)	0.013 (3)	0.005 (3)	0.007 (3)	0.006 (3)
O9	0.011 (3)	0.018 (4)	0.022 (4)	0.005 (3)	0.000 (3)	−0.002 (3)
O10	0.022 (4)	0.023 (4)	0.025 (4)	0.019 (3)	0.013 (3)	0.011 (3)
O11	0.022 (4)	0.022 (4)	0.034 (5)	0.007 (4)	0.012 (4)	0.005 (4)

### Geometric parameters (Å, °)

**Table:**

Dy1—O4	2.401 (7)	I2—O6	1.798 (6)
Dy1—O2^i^	2.408 (7)	I2—O4	1.804 (7)
Dy1—O8^ii^	2.412 (7)	I2—O5	1.812 (7)
Dy1—O6^iii^	2.415 (6)	I3—O9	1.783 (8)
Dy1—O7	2.429 (6)	I3—O8	1.812 (7)
Dy1—O1	2.438 (8)	I3—O7	1.813 (7)
Dy1—O10	2.453 (7)	O2—Dy1^i^	2.408 (7)
Dy1—O5^i^	2.461 (6)	O5—Dy1^i^	2.461 (6)
I1—O1	1.804 (7)	O6—Dy1^iii^	2.415 (6)
I1—O2	1.809 (7)	O8—Dy1^ii^	2.412 (7)
I1—O3	1.814 (7)		
			
O4—Dy1—O2^i^	75.1 (2)	O7—Dy1—O10	126.1 (2)
O4—Dy1—O8^ii^	149.7 (2)	O1—Dy1—O10	151.9 (2)
O2^i^—Dy1—O8^ii^	78.5 (2)	O4—Dy1—O5^i^	112.2 (2)
O4—Dy1—O6^iii^	90.6 (2)	O2^i^—Dy1—O5^i^	73.6 (2)
O2^i^—Dy1—O6^iii^	142.8 (2)	O8^ii^—Dy1—O5^i^	73.6 (2)
O8^ii^—Dy1—O6^iii^	101.7 (2)	O6^iii^—Dy1—O5^i^	142.8 (2)
O4—Dy1—O7	135.1 (3)	O7—Dy1—O5^i^	72.9 (2)
O2^i^—Dy1—O7	141.9 (2)	O1—Dy1—O5^i^	69.4 (2)
O8^ii^—Dy1—O7	75.1 (2)	O10—Dy1—O5^i^	132.9 (3)
O6^iii^—Dy1—O7	70.3 (2)	O1—I1—O2	96.8 (3)
O4—Dy1—O1	69.2 (2)	O1—I1—O3	97.2 (3)
O2^i^—Dy1—O1	111.8 (3)	O2—I1—O3	97.7 (3)
O8^ii^—Dy1—O1	136.0 (2)	O6—I2—O4	99.6 (3)
O6^iii^—Dy1—O1	93.9 (3)	O6—I2—O5	97.8 (3)
O7—Dy1—O1	72.0 (2)	O4—I2—O5	95.5 (3)
O4—Dy1—O10	84.5 (3)	O9—I3—O8	99.4 (3)
O2^i^—Dy1—O10	68.8 (3)	O9—I3—O7	101.4 (3)
O8^ii^—Dy1—O10	72.1 (2)	O8—I3—O7	96.1 (3)
O6^iii^—Dy1—O10	75.9 (3)		

Symmetry codes: (i) −*x*, −*y*−1, −*z*; (ii) −*x*, −*y*−1, −*z*−1; (iii) −*x*+1, −*y*−1, −*z*.

### Hydrogen-bond geometry (Å, °)

**Table:**

*D*—H···*A*	*D*—H	H···*A*	*D*···*A*	*D*—H···*A*
O10—H10A···O3^iv^	0.80	2.29	2.873 (10)	131
O10—H10B···O9^iv^	0.80	2.33	2.753 (11)	114
O11—H11A···O8^v^	0.80	2.22	2.954 (11)	153
O11—H11B···O7^i^	0.80	2.26	2.946 (11)	145

Symmetry codes: (iv) *x*, *y*−1, *z*; (v) *x*, *y*−1, *z*+1; (i) −*x*, −*y*−1, −*z*.
